# An Update on Sexual Transmission of Zika Virus

**DOI:** 10.3390/pathogens7030066

**Published:** 2018-08-03

**Authors:** Hercules Sakkas, Petros Bozidis, Xenofon Giannakopoulos, Nikolaos Sofikitis, Chrissanthy Papadopoulou

**Affiliations:** 1 Microbiology Department, Faculty of Medicine, School of Health Sciences, University of Ioannina, 45110 Ioannina, Greece; cpapadop@uoi.gr; 2 Department of Urology, Faculty of Medicine, School of Health Sciences, University of Ioannina, 45110 Ioannina, Greece; xgiannakop@cc.uoi.gr (X.G.); nsofikit@cc.uoi.gr (N.S.)

**Keywords:** Zika virus, flavivirus, *Aedes*, Host-virus interactions, testis, sexual transmission, epidemics

## Abstract

Zika virus (ZIKV) is a single-stranded RNA virus belonging to the arthropod-borne flaviviruses (arboviruses) which are mainly transmitted by blood-sucking mosquitoes of the genus *Aedes.* ZIKV infection has been known to be rather asymptomatic or presented as febrile self-limited disease; however, during the last decade the manifestation of ZIKV infection has been associated with a variety of neuroimmunological disorders including Guillain–Barré syndrome, microcephaly and other central nervous system abnormalities. More recently, there is accumulating evidence about sexual transmission of ZIKV, a trait that has never been observed in any other mosquito-borne flavivirus before. This article reviews the latest information regarding the latter and emerging role of ZIKV, focusing on the consequences of ZIKV infection on the male reproductive system and the epidemiology of human-to-human sexual transmission.

## 1. Introduction

Zika virus (ZIKV) is an emerging mosquito-borne flavivirus, transmitted mainly by vectors of the *Aedes* (*Stegomyia*) genus (*Culicidae* family). It was initially isolated in 1947 from the serum of a febrile rhesus monkey, trapped in the canopy of the Zika Forest in Uganda, and one year later from a lot of *Ae. africanus* in the same location [1]. ZIKV circulation was reported sporadically within Africa and Southeast Asia for at least half a century before the 2007 emergence in Micronesia and the virus appearance in 2013 in French Polynesia, where it caused significant outbreaks [2,3,4]. According to the World Health Organization (WHO), in the ZIKV country classification scheme since 2013 through February 2017, there have been 31 countries and territories that have reported cases of central nervous system malformations associated with ZIKV infection [5]. The emergence and significance of ZIKV spread was demonstrated during the 2015 outbreak in Brazil which was estimated to have affected 440,000 to 1.3 million people [5,6]. ZIKV infection is frequently asymptomatic, but also it may be presented as a febrile self-limited disease, characterized by clinical symptoms and signs such as mild fever, headache, rash, arthralgia, myalgia, and conjunctivitis, similar to those caused by dengue (“dengue-like” syndrome) and chikungunya viruses [7]. In addition, it has been also documented that certain neurologic manifestations, including Guillain–Barré syndrome and microcephaly in newborns, initially observed in French Polynesia’s and Brazil’s outbreaks respectively, have a ZIKV origin [8,9]. In the absence of specific antiviral drugs or a vaccine, the treatment is symptomatic and the prevention is limited to individual protection against the mosquitos’ bites and vector control measures [7]. The spread of the virus is facilitated by several factors, including globalization, climate changes, increased human mobility, and increased geographic distribution of arthropod vectors, which may potentially affect more individuals in the future [10].

The natural mode of transmission includes a sylvatic cycle involving hematophagous mosquitoes of the *Aedes* genus and nonhuman primates, and an urban cycle between humans and mosquitoes, most commonly *Ae. aegypti* and to a lesser extent *Ae. albopictus* [11]. Transmission has also been reported during sexual intercourse [12], from infected pregnant mothers to infants during all trimesters and at time of delivery [13,14], and accidentally as a result of a needle-stick injury within a laboratory environment [15]. There are also reports on probable ZIKV transmission during platelet transfusion from seropositive donors to blood recipients [16,17]. Although ZIKV has been isolated from breast milk there is no evidence for a breastfeeding transmission mode [18]. In addition, ZIKV has been detected in amniotic and seminal fluids, saliva and urine, increasing the potential of alternative sources of nonvector-borne transmission of the disease [19]. Nevertheless, sexual transmission is reported to be the most important route of nonvector-borne ZIKV mode of transmission, in view of the fact that it is associated with increasing risk of further virus distribution to nonendemic areas [20], including southern states of the USA, northern Australia and southern Europe, where mosquitoes of the *Aedes* genus can breed [21]. Zika is a disease of major public health importance, and in this review we aim at providing an update of the existing published data on ZIKV sexual transmission, with particular emphasis on highlighting the virus–host interactions, and presenting the available data on animal model studies, as well as the epidemiology and clinical manifestations of the disease.

## 2. Host–Virus Interactions

### 2.1. Tropism

**Figure 1:**
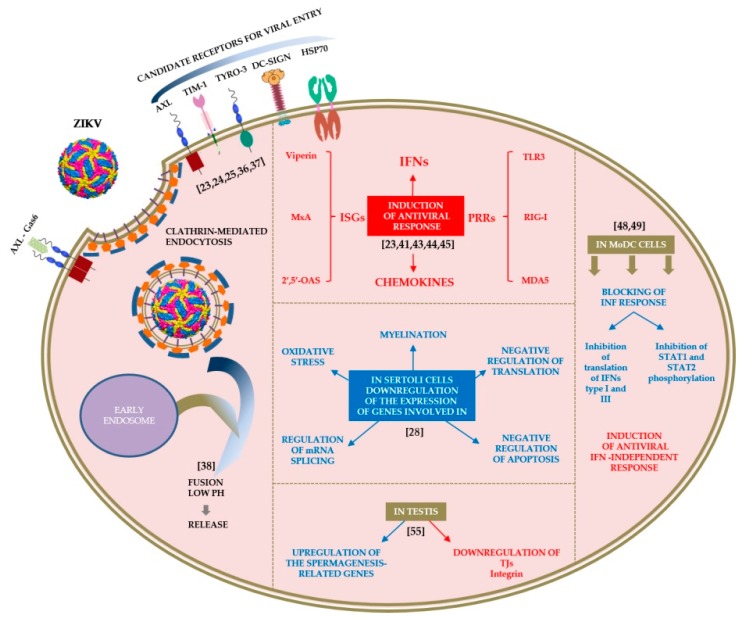
ZIKV attachment and entry into cells and impact of virus–cell interactions on the transcriptome. Rows and legends with red color correspond to upregulated genes while rows and legends with blue color correspond to downregulated genes. The potent interaction of ZIKV virion with the activated homodimeric state of AXL receptor through the Gas6 ligand is also depicted in the scheme [26].

ZIKV can infect and replicate in various human organs and cell types [11]. In the male mouse reproductive tract, ZIKV infection has been detected in several cell types including spermatogonia, primary spermatocytes, Sertoli cells, peritubular myoid cells, Leydig cells, and epithelial cells of the lumen [22]. Among the reported host cell surface molecules that allow the adhesion and entry of the virus are the AXL receptor tyrosine kinase (AXL), the dentritic cell-specific intercellular adhesion molecule 3-grabbing non-integrin (DC-SIGN), the T-cell immunoglobulin and mucin domain 1 (TIM-1), and the Tyro3 protein tyrosine kinase (Tyro3) surface proteins [23,24,25]. The membrane protein AXL is predominantly involved in the infection of various cell types including glioblastoma, epithelial, microglial and human Sertoli cells [26,27,28,29]. It has been reported that knockdown of AXL expression by clustered regularly interspaced short palindromic repeats (CRISPR) interference in HeLa cells and U87 glioblastoma cells prevented the infection by ZIKV [30,31]. In contrast, ablation of AXL in the mouse eye and brain and in human neural progenitor cells does not affect ZIKV infection, suggesting that additional factors may be required for ZIKV entry [32,33]. TIM and additional Tyro3-AXL-Mertk (TAM) family receptors (Tyro3) mediate the ZIKV entry into skin fibroblasts, endothelial cells and retinal cells [23,34,35]. Recently, in vitro binding assays have confirmed a significant degree of binding between Zika envelope protein (ZENV) and both TIM-1 and heat shock protein 70 kDa HSP70 [36]. However, incubation of Sertoli cells with blocking antibodies against AXL reduces viral infection, while inhibition of TIM-1 and TIM-4 receptors, and TAM receptors (Tyro3 and Mertk) did not significantly affect viral replication [28]. Interestingly, it appears that Tyro3 receptors primarily expressed on the mid-piece of human spermatozoa, play a role in ZIKV-binding while TIM-1 plays a more prominent role than AXL in placental cells [34,37]. In a more recent study it is reported that ZIKV entry into the host cells also requires clathrin-mediated endocytosis and a low pH for membrane fusion [38]. These data suggest that depending on the cell type, either different co-receptors are needed for virus entry or that the restriction point is further downstream of receptor binding (Figure 1).

### 2.2. Cell Interactions

There are limited data on ZIKV interactions with human semen cell types to shed enough light on the mechanisms of sexual transmission and on the remarkable viral persistence in semen after remission of clinical signs [22,37,39]. The existing knowledge on virus–cell interactions is that ZIKV like other viruses, upon entering the host cells interferes with the cellular metabolism, disrupting the cell cycle progression and triggering the host immune response. In a recent review article by Wang et al. (2017) regarding the molecular host responses underlying ZIKV infection it is reported that the virus activates transcription factors which cause dysregulation of host cell transcription, leading to antiviral and pro-inflammatory responses, apoptosis and altered cell proliferation [40]. Experimental data supporting the activation of these modules, come from several publications on the transcriptional responses induced by ZIKV [23,41,42,43,44]. Although in these reports different tissue/cell types were used, the common finding is that ZIKV infection induces a strong antiviral response that includes upregulation of interferon stimulated genes (ISGs) and chemokines. Specifically, ZIKV infection increased the expression of Toll-like receptor 3 (TLR3), retinoic acid-inducible gene I (RIG-I) and melanoma-differentiation factor 5 (MDA5) RNA in human fibroblasts [23]. These molecules belong to pattern recognition receptors (PRR) that recognize pathogen-associated molecular patterns (PAMP) and are activated as well by other flaviviruses such as Dengue virus (DENV) [45]. The activation of PRRs, is the first step in the cascade of an antiviral response which includes the expression of ISGs and type I and type III interferons (IFNs). A significant induction in the IFNs response at the level of IFN-β, IFN-λs and ISGs such as Viperin, MxA and 2′,5′-OAS was observed when human monocyte-derived dendritic cells (MoDCs) were challenged with ZIKV [46]. It should be noted that the type III IFN (IFN-Λ1/2/3) response is crucial for the antiviral activity against a panel of viruses in a very narrow subset of cells and tissues including the epithelial surfaces of the female genital tract [47]. Interestingly, there is also experimental data from ZIKV-infected human MoDCs from healthy donors, or myeloid dendritic cells (mDCs) from female patients with acute ZIKV infection indicate a ZIKV–dependent downregulation of the ISGs expression [48,49]. According to the study by Bowen et al. (2017), ZIKV infection of MoDCs induces the transcription of the type I IFN-related genes, with only minimal translation of type I or III IFN proteins detected. The authors also concluded that in this cell type, ZIKV may subvert the type I IFN responses through a combination of direct antagonism of the IFN translation, and downstream blocking of STAT1 and STAT2 phosphorylation, while the virus may induce an antiviral state through an IFN-independent activation of the RIG-I-like receptors (RLRs) signaling pathway [48].

RNASeq (RNA sequencing) analysis of infected Sertoli cells revealed that acute ZIKV infection results in massive dysregulation of host transcripts (>9000) [28]. Specifically, cytokine fibroblast growth factor 2 (FGF2) was found to be the most upregulated transcript, which along with the glial cell line-derived neurotrophic factor (GDNF) contributes to spermatogonial stem cell (SSC) maintenance and spermatogenesis [28]. FGF2 is known to inhibit apoptosis and to promote virus replication [50,51]. The same work in Sertoli cells also revealed downregulation of mRNAs that encode proteins involved in response to oxidative stress, regulation of mRNA splicing via spliceosome, myelination, negative regulation of translation and apoptosis [28]. In ZIKV-infected human fetal neural stem cells (fNSCs), the cooperative action of the viral proteins NS4A and NS4B can suppress Akt-mTOR signaling leading to the upregulation of autophagy [52]. On the host edge, autophagy is one of the processes involved in the maintenance of cellular homeostasis and the control of infection. ZIKV, like other flaviviruses, induces cellular autophagy pathways in several cell types and uses them to benefit its life cycle [22,53,54]. By exerting pleiotropic effects on crucial survival and homeostasis mechanisms, ZIKV may also induce apoptosis in human neural progenitor cells (hNPCs) through p53-mediated caspase-3 activation [43]. In order to understand the effects caused by ZIKV infection on testes, researchers isolated testes from ZIKV infected mice at 5 dpi or control mice and subjected them to transcriptome analysis. The gene ontology analysis revealed the upregulation of gene associated with spermatogenesis and the downregulation of genes associated with tight junctions (TJs) and integrin [55]. The results indicate that ZIKV infection may alter the morphological architecture and function of testis, in a way favoring the sexual transmission of the virus (Figure 1). Hence, what type of virological changes induce increased viral pathogenicity or which viral proteins are responsible for the enhanced pathogenicity and how the recently emergent ZIKV strains alter their interactions with the host cells remains to be resolved by future research.

### 2.3. Laboratory Animals

The current murine models used for studying the virus–host interactions are particularly important for understanding the mechanism of ZIKV sexual transmission. These models have been used to address questions regarding the long-term persistence of the virus in the testis and semen, the replication of the virus in the vaginal canal and the fetal risk via sexual exposure to ZIKV. Persistent shedding of ZIKV RNA in male genital tract tissues and bodily fluids, such as urine and semen, has been documented months after the onset of symptoms [56,57]. In humans, ZIKV can overcome interferon signaling either through proteasomal degradation or through inhibition of the phosphorylation of signal transducer and activator of transcription proteins 1 and 2 (STAT1 and STAT2) [48,58]. Therefore, similar to DENV, in order to study ZIKV pathogenesis, murine models in which the IFN response was deficient have to be used [59]. Experimental work in these murine models has confirmed some of the aforementioned observations related to humans. In particular, high levels of ZIKV RNA and infectious viral particles were detected in the testis and epididymis of infected wild-type C57BL/6 mice which had been treated before with a single dose of monoclonal antibody against the IFNα and IFNβ receptor 1 (Ifnar1) [22]. Similar results were demonstrated in ZIKV MEX-infected IFNAR1^−/−^ mice where the authors observed testicular atrophy with decreased testosterone production due to active infection of Leydig cells within the interstitial region of the testes [60]. Interestingly, these findings were noticed at 21 dpi when there was no detectable ZIKV RNA in the mice blood, suggesting that the virus could establish a persistent infection within the testes as it was reported in humans. The specific cell types of the reproductive tract that could serve as a reservoir for ZIKV infection is still under debate. Recently, immunochemistry experiments in AG129 and IFNAR^−/−^ mice infected with a Puerto Rican ZIKV isolate showed that infection of the epididymal tubular epithelial cells is the earliest and the most robust, followed days later by infection of cells within the seminiferous tubules [61]. Published data suggest movement of the infection from the epididymis into the seminiferous tubules rather than movement of the virus from testicular interstitial cells across the blood–testis barrier. These observations are in agreement with previous reports in vivo [62,63] in which viral antigen was localized as early as 7 or 10 dpi throughout the seminiferous epithelium including all layers of spermatogenic cells and Sertoli cells but not throughout Leydig cells or myoid peritubular cells as reported by other researchers before [60,64].

**Figure 2:**
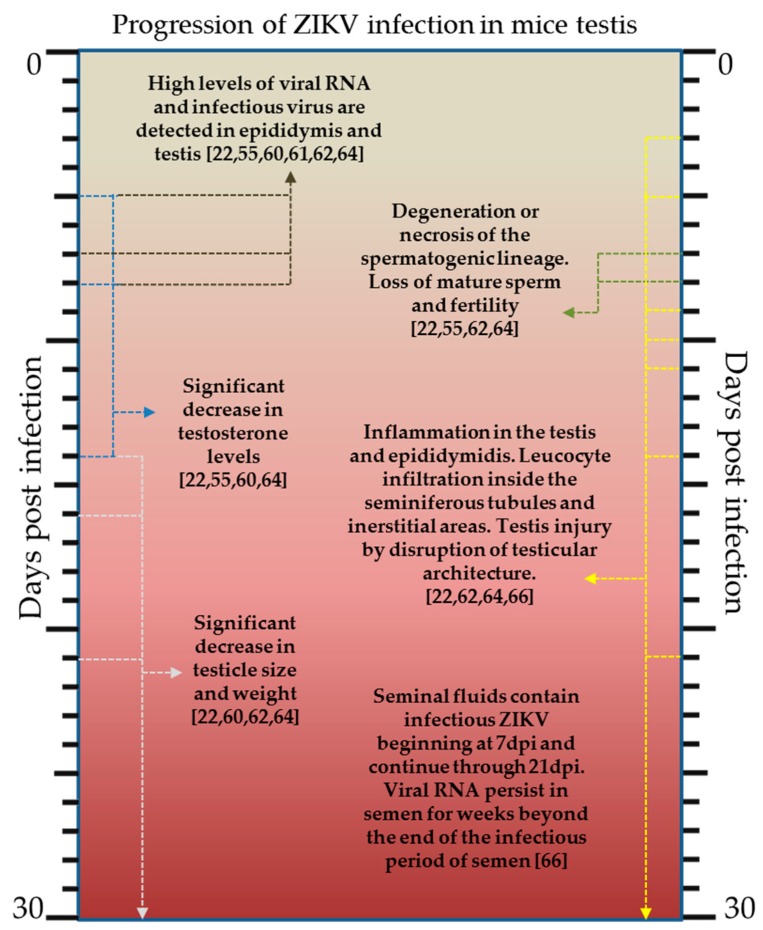
Progression of ZIKV infection in testis of immunodeficient mice. Key findings from several histopathologic analyses, immunofluorescence analyses and real time qRT-PCR analyses in different mouse models are summarized. Horizontal lines indicate the time of infection that each finding has been reported in the corresponding reference.

In ZIKV PRVABC59 infected IFNAR^−/−^ mice at 7 dpi scattered necrosis of epithelial cells was observed in the epididymis while at 14 dpi the seminiferous tubule was completely replaced by necrotic debris and inflammatory cells, a mix of which (including macrophages, neutrophils and lymphocytes) infiltrated the connective tissue areas surrounding the tubules [62]. During the progress of ZIKV infection vacuolar changes in Sertoli cells are observed, which are followed by downregulation in the expression of TJ-associated proteins such as various members of the claudin (Cldn) and occludin (Ocln) families at both RNA and protein levels [55]. The loss of TJ-associated proteins is considered responsible for decreased Sertoli cells barrier (SCB) integrity, enhanced infiltration of macrophages and adaptive immune cells into the lumen. Thus, ZIKV infection and the release of inflammatory mediators from the immune cells which cross the barrier, along with those released from infected Sertoli cells and possibly Leydig and peritubular cells, could cause damages to the testis [49]. The consequence of such significant injury to the testis of ZIKV infected Rag1^−/−^ C57BL/6 mice was cell death by apoptosis in the seminiferous tubules and lumen of the epididymis resulting in decreased testosterone and inhibin B production, and oligospermia [22]. Although variable orchitis and epididymitis has been observed in different mice models, there are still conflicting reports concerning the infection of accessory sex glands which may constitute a possible site of virus persistence within the male reproductive tract [61,64]. Vasectomized male AG129 mice that were inoculated intraperitoneally with ZIKV could transmit the virus through sexual transmission suggesting that testes are not the only source of infection [65]. In the same study, non-vasectomized male AG129 mice were able of ZIKV sexual transmission for a period starting 7 days post-inoculation through 21 days post-inoculation, although the viral RNA persisted in semen for weeks after the end of the semen infectivity period (Figure 2). The mice models provide a powerful tool to study the pathogenesis of ZIKV infection, yet more research is needed to fully understand the pathogenesis and consequences of the sexually transmitted ZIKV infection [66].

## 3. Epidemiology

Until the most recent global outbreak and from 1947 to 2007, only rare febrile self-limited cases were reported in African and Southeast Asian regions [10]. In 2007, ZIKV spread outside Africa and Asia, and the first epidemic (an estimated 5000 individuals were infected) was recorded in Yap Island, Federated States of Micronesia, North Pacific [2]. Six years after ZIKV was detected in Yap Island, an outbreak occurred in French Polynesia, South Pacific, involving about 10% of the population [3], and complicated with neurological manifestations defined as Guillain–Barré syndrome [7].

As per the latest World Health Organization (WHO) situation report by March of 2017, 84 countries, territories or subnational areas have reported vector-borne ZIKV transmission, 31 of them reporting microcephaly and other central nervous system (CNS) malformations, 23 recording an increased incidence of Guillain–Barré syndrome and 13 reporting evidence of person-to-person ZIKV transmission; seven from Europe (France, Germany, Italy, Netherlands, Portugal, Spain, United Kingdom), five from the Americas (Argentina, Canada, Chile, Peru, USA), and one from the Pacific region (New Zealand) [67]. Among newborns, the incidence of ZIKV-related congenital malformations such as severe microcephaly has been estimated from 1% (French Polynesia), to 6% (USA), and up to 46% (Brazil) [68]. In 2015–2016, 2656 confirmed cases of neurological abnormalities were detected, most of which (89%) were diagnosed in Brazil [69]. Prior to the last ZIKV epidemics, cases of nonvector flaviviruses’ transmission were rarely reported and they were, most often referring to DENV infection [20]. Up to date sexual transmission of other arboviruses has never been documented in the existing literature [12].

The first case of sexually transmitted ZIKV infection occurred in Colorado, USA, in 2008. A female patient with no previous travel history to any endemic region, developed symptoms related to ZIKV infection. The patient mentioned that she had vaginal sexual intercourse with her infected husband as soon as he had returned home from Senegal, just before the onset of his clinical manifestations [12]. Since then, the number of sexually acquired ZIKV cases in nonendemic regions has increased, affecting individuals without recent travel or residential history to endemic countries, who were infected by their sexual partners arriving from endemic areas [70]. The fact that almost 80% of the patients are usually asymptomatic, increases the chances of getting infected by sexual contact [70], thus strengthening the dynamics of ZIKV distribution [71]. Hence, the public health hazards and health impacts arising from the sexual mode of transmission may lead to extensive spread of the ZIKV, expanding to several geographical regions and to healthy human populations, not excluding the implications on the fertility and safety of sperm and ovum banks [72]. Therefore, a sexual transmission framework was developed in 2017 by the WHO, in order to evaluate the dynamics of the transmission mode and its epidemic potential [73].

According to a mathematical model developed by Maxian et al. (2017), the sexual transmission mode appears not to act as a determinative factor in endemic areas with high mosquito density [74]. However, during the last epidemic in Rio de Janeiro, the incidence of ZIKV in sexually active females, accounted for 90% higher than for adult men, excluding pregnant women, thus indicating significant relation of the sexual transmission route to ZIKV infection [21]. In a systematic review conducted by Moreira et al. (2017), a total of 18 studies and 27 probable or laboratory-confirmed sexually acquired cases were included, in which the infection was transmitted primarily from male to female (92.5%), and to a lesser extent from male to male (3.7%) and from female to male (3.7%). The main mode of transmission was unprotected vaginal contact (96.2%), followed by oral (18.5%) and anal (7.4%) intercourses [70]. Subsequently, the sexually transmitted ZIKV disease is likely to have spread further, and by the first trimester of 2018, the Centers for Diseases Control and Prevention (CDC) had reported for the USA a total of 45 cases in 2016 and seven cases of sexually acquired ZIKV in 2017 [75].

## 4. Clinical and Laboratory Findings

In most cases, the infected patients who transmitted ZIKV to their sexual partners were symptomatic, however cases of asymptomatic male-to-female sexual transmission via vaginal and oral intercourses, have also been reported [71,76]. Considering that the male-to-female sexual transmission rates are significantly high, the risk of ZIKV vertical transmission in pregnant women and its complications in fetuses such as microcephaly, is remarkably high too [70]. Apart from the above mentioned clinical findings of the disease, in men ZIKV infection is rarely manifesting as microscopic or macroscopic hematospermia and prostatitis characterized by mild dysuria and perineal pain [12,70]. Presence of white blood cells in semen as a marker of inflammation and affected semen concentration has been demonstrated in ZIKV male patients [70].

ZIKV has been isolated from body fluids of the urogenital system such as semen, urine, vaginal fluid and cervical mucus [11,77]. Real-time reverse-transcription polymerase chain reaction (rRT-PCR) is usually used for ZIKV RNA detection in semen, while ZIKV may also be identifiable by its effects in cell cultures [15,78]. According to several studies, ZIKV RNA has been detectable in semen for up to 188 days after the onset of clinical symptoms, while the persistence in urine, serum, cerebrospinal fluid, saliva and vaginal secretions has been detected for 91, 34, 7, 91 and 14 days respectively, indicating that ZIKV persists for longer time in seminal fluid, probably replicating in testicles or seminal glands [11,70,79,80]. ZIKV viral load has been found 10^5^ times higher than the detected levels in both urine and blood for more than two weeks after the clinical symptoms onset, indicating replication in the male genital tract [81]. In addition, infectious virus has been detected in semen up to 69 days after the onset of clinical manifestations [82] and ZIKV RNA up to 62 days in serum of pregnant women [14].

Considering that ZIKV may persist in sperm for a long time after clinical recovery and the incubation time ranges between 3 and 12 days, there is strong evidence of a delayed male-to-female sexual transmission, ranging from 32 to 41 days after the onset of clinical signs in a male partner [83]. However, it is difficult to prove whether ZIKV is potentially infectious, since ZIKV RNA has been detected in semen of men with azoospermia or in seminal fluid without spermatocytes because of vasectomy or centrifugation [15]. According to CDC recommendations both symptomatic and asymptomatic male patients or travelers returning from areas with high risk of ZIKV infection should have safe sexual practice for at least six months, while infected female partners or women returning from an endemic area should wait at least eight weeks before considering pregnancy [84].

## 5. Management and Prevention

Sexual transmission of ZIKV is one of the nonvectorial transmission routes of the virus, the others being through breast feeding, saliva, urine, blood and plasma-derived medicinal products, and vertical/transplacental spread [12,13,14,15,16,17,18,19]. However, the main mode of ZIKV transmission is through mosquito bites [10,11,15]. Τhus, for an effective management of the disease, control strategies have to be focused on both vectorial and nonvectorial transmission modes, and on development of reliable and efficient diagnostics, therapeutics and vaccines.

Strategies for vectors’ management include mechanical, chemical and biological control measures targeting at mosquito population control. Such measures include inhibition of mosquitoes reproduction through reduction of their breeding sites and eradication of the larvae and adults using: chemical insecticides with larvicidal and adulticidal action, insect growth regulators (IGRs), chitin synthesis inhibitors, release of sterile male mosquitoes, use of bacterial biolarvicides (*Bacillus thuringiensis* subsp. *israelensis*, *Bacillus sphaericus*) and fungal adulticides (*Metharhizium anisopliae, Beauveria bassiana, Aspergillus nomius*), use of certain species of mosquitoes and copepods which feed on *Aedes* sp. larvae and adults, tadpoles, predatory fish species and plant derived products with insecticidal activities, genetically modified mosquitoes transmitting lethal genes to their offspring, and last but not least reducing exposure to mosquito bites by applying personal protection measures such as window/door nets, proper clothing and insect repellents [85,86].

The management of sexually transmitted ZIKV has to be focused on proper information and awareness of the community on safe sex practices in particular in endemic areas. Advice on safe sex (use of condom) and avoidance of unprotected sexual intercourse when visiting high risk areas are precautionary measures of outmost importance [87]. Travelers from endemic countries should be under close watch for any symptoms of the disease for at least one month after returning home and family planning after visiting endemic areas has to be postponed for at least four weeks (two weeks for the incubation period plus two weeks for the end of viremia) [88]. Susceptible subjects should be advised to avoid sexual intercourse for up to six months from the onset of symptoms in male partners or from the time of the disease diagnosis [89]. Also, the risk of transfusion recipients infecting their sexual partners is of major concern and blood safety practices have to be re-evaluated in relation to ZIKV emergence [90]. The long-term management and prevention of sexually transmitted ZIKV requires good knowledge and understanding of the reproductive and sexual behavior of men and women in endemic communities. A recent social study contacted in Iquitos, Peru, among males and females revealed negative feelings for condoms and inconsistent use of contraception [91]. The described sexual behavior patterns signify the importance of sexual education as an effective intervention in ZIKV control.

The most effective control of infectious diseases is vaccination and currently there are a number of ZIKV vaccine candidates under development including the following categories of vaccine platforms: purified inactivated, live attenuated, recombinant subunit, nucleic-acid-based (DNA, mRNA self-replicating RNA), viral-vectored, cytotoxic T lymphocyte vaccines, Lysosome-associated membrane protein vaccines [92,93,94,95]. According to the WHO, there is need for two ZIKV vaccines: a vaccine for emergency outbreak response, suitable for mass vaccination during an epidemic or outbreak to protect women of reproductive age and prevent congenital Zika syndrome, and a vaccine for immunization of the general population in endemic areas during the interepidemic periods, administered from early childhood to adults [93].

Since the emergence of ZIKV, more than 40 vaccine candidates using different platforms and protection mechanisms have been reported to be under clinical trials. Although their number is remarkable, the licensure of a safe and effective ZIKV vaccine is not expected soon due to several reasons, mainly because of difficulties in the implementation of phase III trials. In particular the spatial and temporal heterogeneity of ZIKV transmission, the variety of clinical signs, the lack of reliable diagnostic tests and the unpredictability of the epidemics make the implementation of phase III clinical trials difficult [92,93]. So far, the vaccine efficacy trials are confined to either murine or non-human primate animal models. The development of vaccines able to prevent vertical transmission and the congenital syndrome is very challenging but clinical trials for testing vaccine efficacy in relation to fetus protection are going to take time and may have high costs to be completed. Hence, currently there is no commercially available vaccine against ZIKV, despite the promising outcomes reported worldwide by several research groups.

## 6. Conclusions

For over half a century, ZIKV circulation was associated with sporadic reports of cases of a febrile self-limited disease, transmitted by mosquitoes of the *Aedes* genus. The infection-related Guillain–Barré syndrome and neonatal microcephaly, raised concerns of further spread of the virus, due to other modes of transmission, in particular sexual transmission. After the first sexually acquired ZIKV infection was recorded, the number of sexually transmitted cases has increased in nonendemic regions where the laboratory diagnosis is accessible. The sexually acquired infection is transmitted primarily from male to female by unprotected vaginal contact and ZIKV RNA has been isolated from semen, urine, vaginal fluid and cervical mucus. Mouse models have provided significant insights on the molecular biology and clinical manifestations of ZIKV infection of the male reproductive tract. Still, there are conflicting results which must be clarified in order to identify critical parts of the urogenital system (organs, accessory glands) in ZIKV pathogenesis. Uncertainties related to which phase of spermatogenesis sperms are vulnerable to ZIKV infection or why, in the presence of anti-ZIKV antibodies, infected men are still able to transmit the virus to their sexual partners have to be resolved. Also, further work targeting specific cell types within the male reproductive system is needed in developing effective antiviral therapies. The fact that ZIKV can be detected in seminal fluids several weeks after infection and the lack of adequate knowledge on the virus pathogenesis in relation to sexual transmission pose unanswered albeit important questions that demand further exploration. Conclusively, the emerging research evidence on the unique ability of ZIKV to spread through sexual transmission underlines the importance of the public health guidelines and continuous vigilance regarding sexually transmitted diseases.
